# Antidiabetic Activity of Benzopyrone Analogues in Nicotinamide-Streptozotocin Induced Type 2 Diabetes in Rats

**DOI:** 10.1155/2014/854267

**Published:** 2014-12-04

**Authors:** Yogendra Nayak, Venkatachalam Hillemane, Vijay Kumar Daroji, B. S. Jayashree, M. K. Unnikrishnan

**Affiliations:** ^1^Department of Pharmacology, Manipal College of Pharmaceutical Sciences, Manipal University, Manipal, Karnataka 576104, India; ^2^Department of Chemistry, Kasturba Medical College International Centre, Manipal University, Manipal, Karnataka 576104, India; ^3^Department of Pharmaceutical Chemistry, Manipal College of Pharmaceutical Sciences, Manipal University, Manipal, Karnataka 576104, India; ^4^College of Pharmacy, Anjarakandy Integrated Campus, Kannur, Kerala 670612, India; ^5^Department of Pharmacy Practice, Manipal College of Pharmaceutical Sciences, Manipal University, Manipal, Karnataka 576104, India

## Abstract

Benzopyrones are proven antidiabetic drug candidate in diabetic drug discovery. In this view novel synthetic benzopyrone analogues were selected for testing in experimental diabetes. Type 2 diabetes (T2D) was induced in Wistar rats by streptozotocin (60 mg/kg, i.p.) followed by nicotinamide (120 mg/kg i.p.). Rats having fasting blood glucose (FBG) >200 mg/dL, 7 days after T2D-induction, are selected for the study. Test compounds and standard treatment were continued for 15 days. FBG, oral glucose tolerance test (OGTT), and insulin tolerance test (ITT) were determined on 21st day after induction of T2D. Plasma lipids and serum insulin were estimated. Homeostatic model assessment (HOMA-IR) was then calculated from serum insulin. Rats were sacrificed and pancreas was isolated for histopathological observations. Oxidative stress markers were estimated in liver homogenate. Quercetin, a natural product with benzopyrone ring, showed significant hypoglycemic activity comparable to glibenclamide. Treatment with test compounds lowered the FBG and insulin resistance was significant alleviated as determined by OGTT, HOMA-IR, and ITT. There was significant normalisation of liver antioxidant enzymes compared to diabetic rats indicating that all the synthesised benzopyrone analogues are beneficial in reducing oxidative stress and are on par with the standard quercetin and glibenclamide in experimental T2D.

## 1. Introduction

Type 2 diabetes mellitus (T2D) is a disorder characterized by insulin resistance and pancreatic *β*-cells dysfunction. The combination of glucotoxicity and lipotoxicity termed as “glucolipotoxicity” is said to be the cause for *β*-cell dysfunction in T2D [1]. The four major molecular mechanisms implicated in chronic hyperglycemia-induced tissue damage are (1) activation of PKC and DAG pathway, (2) hexosamine pathway, (3) AGEs formation, and (4) polyol pathway [2]. The lipid peroxidation of vascular wall due to oxidative stress in hyperglycaemia is the major cause for diabetic microvascular disease [3]. Currently available drugs reduce blood glucose level either by increasing the tissue uptake or by increasing the insulin secretion. There is no successful drug to treat the metabolic disorders associated with diabetes [4].

Benzopyrone is the pharmacophore present in the naturally occurring plant products such as flavonoids and coumarins. The benzopyrone scaffold has been tried for treatment of many diseases. The natural products with the *γ*-benzopyrone scaffolds especially flavonoids are explored greatly for treatment of diabetes [5]. Although such compounds cannot cure DM because of the complex nature of disease and genetic involvements, they can ameliorate some of the consequences of DM related metabolic complications. In DM the flavonoids have been reported to act at different steps of glucose digestion till its disposition [6]. Previous reports from our department revealed herbal extracts have antidiabetic activity and the major constituent responsible was a flavonoid [7, 8].

**Figure 1:**
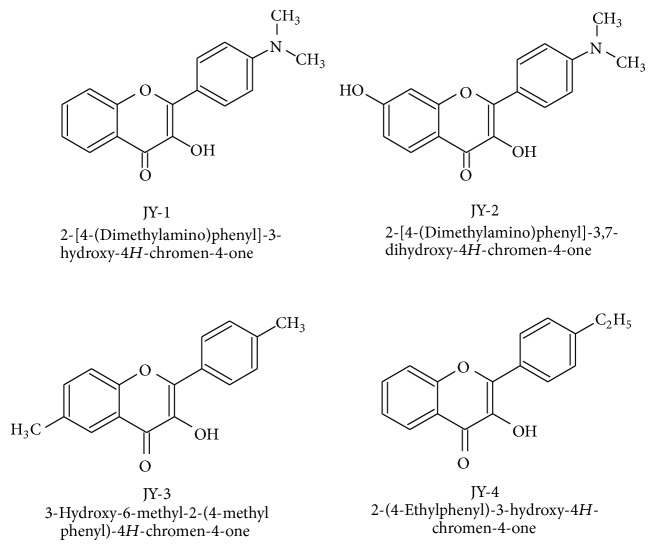
Structures of selected benzopyrone analogues (JY-1, JY-2, JY-3, and JY-4).

The major limitations for natural flavonoids were cost of purification from the natural sources as well as potency, efficacy, solubility and stability. These factors shifted the idea of many drug discovery teams to go for synthetic alternatives. The flavonoids in general have the common ring *γ*-benzopyrone (or benzopyran-4-one), substituted with phenyl group at position 2 [9]. Synthetic *γ*-benzopyrone derivatives, with a structure similar to natural flavones, are more likely to offer better lead molecules because antidiabetic properties are known to be linked to antioxidant and antiglycation potential. Therefore we have selected *γ*-benzopyrone for structural modification to obtain better analogues. We have chosen four test compounds (Figure 1) which could be prepared easily, using a simple two-step synthetic procedure, facilitating better yield and cost effective synthesis [10, 11].

## 2. Materials and Methods

### 2.1. Chemicals

STZ, quercetin, TBA, BSA, DTNB, GSH, and SOD were purchased from Sigma-Aldrich, USA. Nicotinamide, anthrone, and oyster-glycogen were procured from Himedia, Mumbai, India. CMC was purchased from SD Fine Chemicals, Mumbai, India. Glibenclamide was the gift from Micro Labs Ltd, Bangalore, India. The test compounds and standards were suspended in 0.5% CMC before administration to the animals.

### 2.2. Animals

Male Wistar rats (7 to 8 week; 150–200 g), maintained in sanitized polypropylene cages (3 per cage) in air conditioned rooms (23 ± 2°C, 35–60% humidity with 12 h light-dark cycle), were obtained from the central animal facility of Manipal University. The rats were fed with pellet diet and water ad libitum. Prior approval was obtained from the Institutional Animal Ethical Committee (IAEC) (letter number IAEC/KMC/06/2006-2007) and experiments are conducted as per the standard guidelines.

### 2.3. Induction of T2D Diabetes in Wistar Rats and Treatment with Test Compounds

#### 2.3.1. Preparation of 0.1 M Citrate Buffer

Accurately weighed quantity of trisodium citrate (1.49 g) was dissolved in sufficient milli Q water to produce 100 mL and was adjusted to pH 4.5 with HCl.

#### 2.3.2. Preparation of STZ and Nicotinamide Solution

STZ is freely soluble in water and saline, but it is unstable in both. It is stable in 0.1 M citrate buffer. A solution of STZ of appropriate strength (depending on the required total dose per animal) was prepared by dissolving weighed quantity of STZ in freshly prepared ice cold citrate buffer and was administered i.p. in volumes of ~2 mL/kg (dose: 60 mg/kg). STZ was freshly prepared because it is unstable. Nicotinamide was dissolved in normal saline to yield a strength that is appropriate for administration.

#### 2.3.3. Induction of Diabetes and Treatment

Diabetes was induced by STZ (60 mg/kg), 15 min after the intraperitoneal administration of nicotinamide (120 mg/kg) [12]. After 7 days rats showing FBG between 180 and 220 mg/dL were considered diabetic and included in the study. These diabetic rats were further grouped (*n* = 6) and were treated with test/standards for 15 days according to the following protocol:

(G1) healthy rats as normal control (1.0 mL of 0.5% CMC),
(G2) diabetic control (1.0 mL of 0.5% CMC),
(G3) diabetic + glibenclamide (2.5 mg/kg),
(G4) diabetic + quercetin (10 mg/kg),
(G5) diabetic + JY-1 (80 mg/kg),
(G6) diabetic + JY-2 (80 mg/kg),
(G7) diabetic + JY-3 (80 mg/kg),
(G8) diabetic + JY-4 (80 mg/kg).

The dose of test compounds was based on our earlier studies on dose optimisation. We have optimised the dose of test compounds to 80 mg/kg/p.o./day, based on the oxidative biomarker FRAP (ferric reducing ability of plasma) in comparison with quercetin [13]. Male Wistar rats were used for the experiments because females exhibit variations in the diabetogenic response to STZ [14].

### 2.4. Effects of the Test Compounds in Nicotinamide-STZ Diabetic Rats

#### 2.4.1. Percentage of Decrease in Glycemia

This was calculated with reference to day 7, by the formula

(1) %  Decrease  in  glycemia  =FBG on⁡ day 7−FBG on⁡ day 22FBG on⁡ day 1×100

and mean of each group was taken for comparisons.

#### 2.4.2. Fasting Blood Glucose and OGTT

Glucose tolerance was determined by OGTT on day 15 one hour after the last dose. Twelve-hour fasted animals were administered glucose (2 g/kg, p.o.) and blood samples were collected from the tail tip incision at 0, 15, 30, 60, 90, and 120 min after glucose administration. Blood glucose was estimated by using a glucometer (Accu-chek, Roche). The blood glucose concentration was plotted against time and the area under curve was calculated by following formulae:

(2) $$\begin{array}{c} \text{AUC}=\left[\frac{C_{1}+C_{2}}{2}\right]\times \left(t_{2}-t_{1}\right). \end{array}$$

#### 2.4.3. Estimation of Serum Lipids

Blood was collected by puncturing retroorbital plexus after performing OGTT and serum was separated. Serum TG, TC, and HDL were estimated by semi-autoanalyser, Star 21 (Seac Radim group) using diagnostic reagent kit (Aspen Laboratories, Delhi). VLDL and LDL were calculated by the formulae [15]

(3) $$\begin{array}{c} \text{VLDL}=\frac{\left(\text{TG}\right)}{5};\;\;\text{LDL}=\text{TC}-\text{VLDL}-\text{HDL}. \end{array}$$

TC/HDL ratio was calculated as a marker of dyslipidemia.

#### 2.4.4. Estimation of Serum Insulin and HOMA-IR

Serum insulin was estimated using insulin elisa kit for rats (Linco research Inc.). The insulin resistance was measured by HOMA-IR by the following formula [16]:

(4) HOMA-IR=fasting  plasma  glucose  mg/dL  ×fasting  plasma  insulin  μIU/mL×405−1.

#### 2.4.5. ITT

This test was performed by intraperitoneal injection of insulin bolus (4 IU/kg; Actrapid, Novo-Nordisk). Blood glucose was determined (Section 2.4.2) immediately before and 5, 10, 15, 30, 60, 90, and 120 min after the injection. The first-order rate constant for the disappearance rate of glucose from plasma (K-ITT) was estimated from the slope of the regression line of the log plot of blood glucose against time [17].

#### 2.4.6. Estimation of Serum AGEs and Creatinine

The serum AGEs was estimated as per the reported method [18] with modification. Briefly, the fluorescence intensity of serum was recorded at room temperature (excitation 370 nm, emission 440 nm), using blackwell microplates (Greiner Bio One) and a fluorescence plate reader FLX800-TBDI (Bio-Tek Instruments Inc., USA). Data were analysed using in-built GEN5 software. Fluorescence was expressed as the relative fluorescence intensity (relative to plain serum from the normal untreated rats) in arbitrary units (AU).

Serum creatinine was determined using commercially available kit (Aspen Laboratories, Delhi) with an autoanalyzer (Star 21, Seac Radim Group).

#### 2.4.7. Oxidative Stress Markers and Glycogen in Liver

After OGTT, liver was dissected out, and a 10% liver homogenate was prepared with ice-cold saline-EDTA using Teflon glass homogenizer. The homogenate was centrifuged at 10,000 rpm for 10 min (at 4–8°C) using cooling centrifuge (MIKRO 220R, Hettick Lab Technology, Germany). The pellet was discarded and supernatant obtained was used for the estimation of oxidative stress markers in the homogenate (TBARS, GSH, total thiols, GST, catalase, and SOD) and the total protein was estimated by standard methods [7, 8]. The concentration of antioxidant enzymes was expressed in terms of protein content.

Liver glycogen was estimated by anthrone reagent method [19]. The glycogen content was calculated and expressed in mg/g wet weight of the liver.

#### 2.4.8. Histopathology of Pancreas

Soon after sacrificing the animals, pancreas was isolated from each animal, fixed in 10% formal saline. The tissue was then processed and embedded in paraffin for thin section using microtome (Leica Biosystems). These sectioned slices of pancreas tissue were then stained with hematoxilin and eosin. Pancreatic histopathology was then compared among the different treatment groups for protection of regeneration of pancreatic islet cells.

### 2.5. Statistical Analysis

All the data were presented as Mean ± SEM, and statistical analysis was carried out by one way ANOVA followed by Tukey's post-hoc test using GraphPad Prism 6.0 (Demo Version). A value of *P* < 0.05 was considered statistically significant.

## 3. Results

### 3.1. Induction of Diabetes

In spite of protection from nicotinamide, STZ is lethal to animals at slight variations in doses. Therefore we employed an excess number of rats per experiment. We observed ≈15% mortality in rats within one week after nicotinamide-STZ treatment, which increased to a total of ≈30% (21 died out of 70) at the end of experiment. Out of those rats which survived at the end of 1 week after STZ injection, many developed the required level of hyperglycemia (mean FBG ≈200) that mimics T2D in humans. However, ≈10% (7 out of 70) rats did not develop hyperglycemia in the required range.

The surviving rats which developed the required level of hyperglycemia were divided into different treatment groups for screening the synthesised molecules (G2 was diabetic control; G3 to G8 were treatment groups, Section 2.3.3).

### 3.2. Inhibition of Hyperglycemia by Test Compounds

Table 1 represents the % reduction of glycemia after treatment with test compounds. In the diabetic rats (G2), there was significant increase in the blood glucose when compared with healthy rats (G1). Rats of treatment groups showed significant decrease in % glycemia compared to the diabetic control (G2). None of the treated rats became normoglycemic at the end of the study period. JY-2 showed a maximum antidiabetic effect, with 31.15% reduction in glycemia. On the other hand, glibenclamide produced 44.55% reduction whereas JY-1 also produced moderate antidiabetic action.

### 3.3. Effect of Test Compounds on OGTT

**Figure 2:**
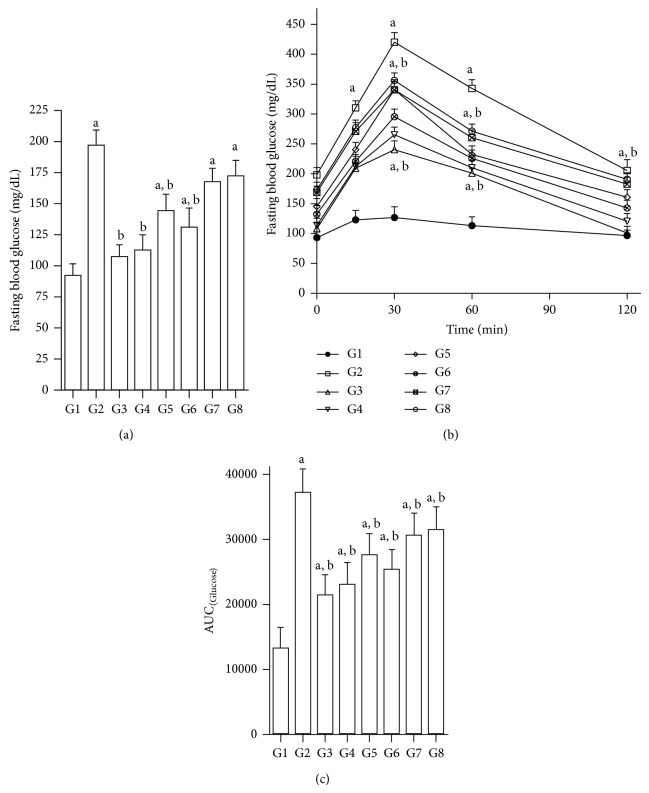
Effect of test compounds on FBG and OGTT in nicotinamide-STZ diabetic rats; (a) fasting blood glucose (FBG) (mg/dL); (b) OGTT; and (c) area under the curve [AUC]; G1: normal control; G2: diabetic control (nicotinamide + STZ); G3: diabetic + glibenclamide (2.5 mg/kg); G4: diabetic + quercetin (10 mg/kg); G5: diabetic + JY-1; G6: diabetic + JY-2; G7: diabetic + JY-3; G8: diabetic + JY-4; (test compounds at optimised dose of 80 mg/kg); ^a^ *P* < 0.05 compared to normal rats (G1); ^b^ *P* < 0.05 compared to diabetic rats (G2) (one-way ANOVA followed by Tukey's post-hoc test).

When normal rats (G1) are challenged with oral glucose (2 g/kg), there was no intolerance when compared to AUC of diabetic rats (G2). Diabetic controls (G2) showed significant elevation in FBG levels and there was significant glucose intolerance. All test compounds (JY-1, JY-2 JY-3, and JY-4) and the standards (quercetin and glibenclamide) produced significant reduction in blood glucose levels over a period of 120 min after glucose challenge (Figure 2). Among the four compounds, JY-2 produced maximum effect, as seen in the previous experiment. JY-2 at dose 80 mg/kg/day was the most potent among the four compounds tested. JY-2 was found to be comparable to quercetin in efficacy (10 mg/kg/day, p.o.) despite statistically significant difference between activity of quercetin and JY-2.

### 3.4. Effects of Test Compounds on Serum Lipids

**Figure 3:**
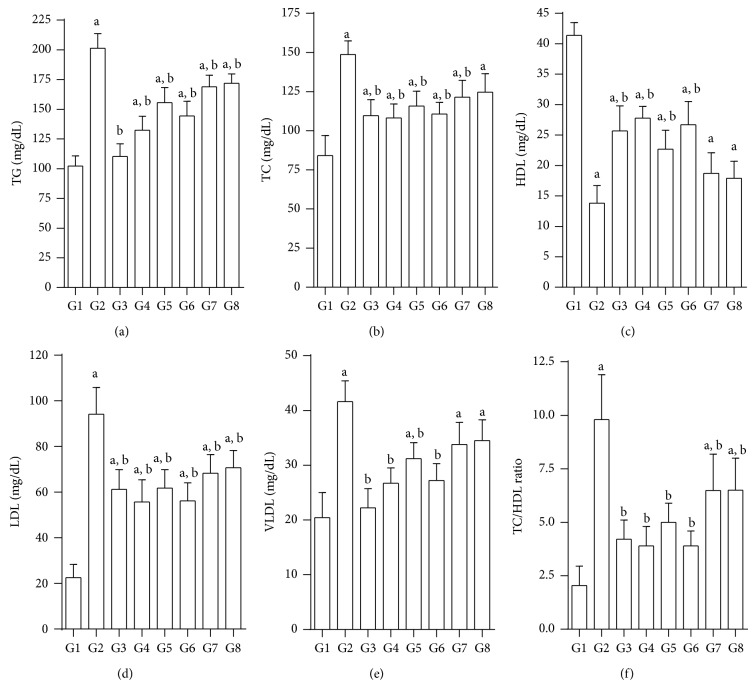
Effect of test compounds on serum lipids in nicotinamide-STZ rats; (a) TG, (b) TC, (c) HDL, (d) LDL, (e) VLDL (f) TC/DHL-ratio; G1: normal control; G2: diabetic control (nicotinamide + STZ); G3: diabetic + glibenclamide (2.5 mg/kg); G4: diabetic + quercetin (10 mg/kg); G5: diabetic + JY-1; G6: diabetic + JY-2; G7: Diabetic + JY-3; G8: diabetic + JY-4; (test compounds at optimised dose of 80 mg/kg); all values mean ± SEM (*n* = 6); ^a^ *P* < 0.05 compared to normal control (G1), ^b^ *P* < 0.05 compared to diabetic control (G2) (one-way ANOVA followed by Tukey's post-hoc test).

Upon induction of diabetes by nicotinamide-STZ, the serum TG, TC, LDL, and VLDL are significantly elevated compared to normal rats (G1). HDL significantly decreased in diabetic controls. Test compounds produced varying effects on different serum lipids. JY-2 was the most effective in lowering TG, TC, and VLDL as well as LDL (Figure 3). This is consistent with previous results. The decline in TC/HDL ratio produced by JY-2 was comparable to that of quercetin and superior to glibenclamide.

### 3.5. Effect of Test Compounds on Serum Insulin, HOMA-IR, and Insulin Tolerance

**Figure 4:**
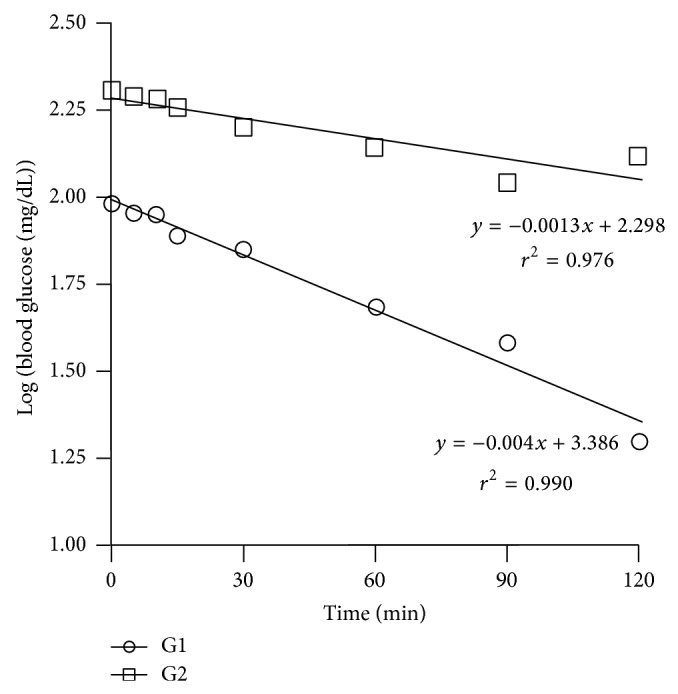
Determination of insulin tolerance (K-ITT) in normal and nicotinamide, STZ diabetic rats; first-order rate constant for the disappearance rate of glucose from plasma (K-ITT) upon insulin treatment (4 IU/kg, i.p.) was estimated from the slope of the regression line of the logarithmic plot of blood glucose (in mg/dL) against time. Slope is negative because blood glucose decreases with time after insulin. (G1: normal rats and G2: diabetes induced rats). The figure does not show the plots of all treated groups because values are close to each other and overlapping.

**Figure 5:**
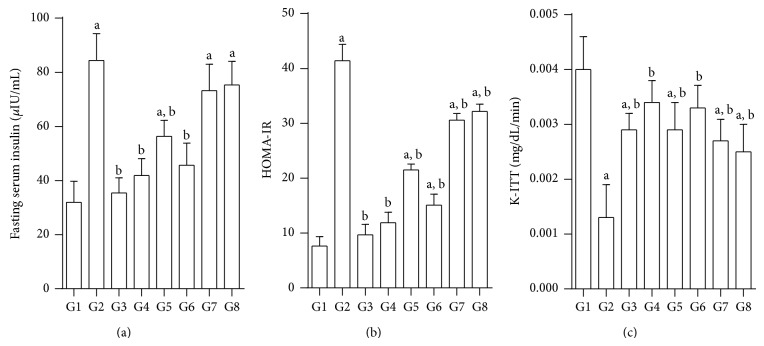
Effect of test compounds on serum insulin, HOMA-IR, and insulin tolerance in nicotinamide, STZ induced diabetic rats; all values mean ± SEM (*n* = 6); (a) fasting serum insulin (*μ*IU/mL); (b) HOMA-IR; and (c) K-ITT (mg/dL/min); G1: normal control; G2: diabetic control (nicotinamide + STZ); G3: diabetic + glibenclamide (2.5 mg/kg); G4: diabetic + quercetin (10 mg/kg); G5: diabetic + JY-1; G6: diabetic + JY-2; G7: diabetic + JY-3; and G8: diabetic + JY-4; (test compounds at optimised dose of 80 mg/kg); ^a^ *P* < 0.05 compared to normal rats (G1), ^b^ *P* < 0.05 compared to diabetic rats (G2) (one-way ANOVA followed by Tukey's post-hoc test).

HOMA-IR is the biomarker that is often used to assess insulin resistance. The basal insulin level increased from 31.93 ± 3.9*μ*IU/mL (controls) to 84.49 ± 5.3 in diabetic rats. There was significant elevation in HOMA-IR in diabetic controls (G2) indicating induction of insulin resistance. Upon treatment with JY-2, there was a significant reduction in HOMA-IR, which was comparable to quercetin treatment. Insulin administration (4 IU/kg, i.p.) to normal (G1), diabetic (G2), and treated animals (G3–G8) produced a decrease in blood glucose levels. However, the rate of decrease in blood glucose was significantly lower in normal rats compared to the diabetic rats (Figure 4). First-order rate constants for the disappearance rate of glucose from plasma (K-ITT) upon administration of insulin (4 IU/kg, i.p.) was estimated as the slope of regression line of logarithmic plot of blood glucose (in mg/dL) against time. Treatment with test compounds produced a significant reversal in glucose clearance in all the groups (Figure 5).

### 3.6. Effect of Test Compounds on Serum AGEs and Creatinine

**Figure 6:**
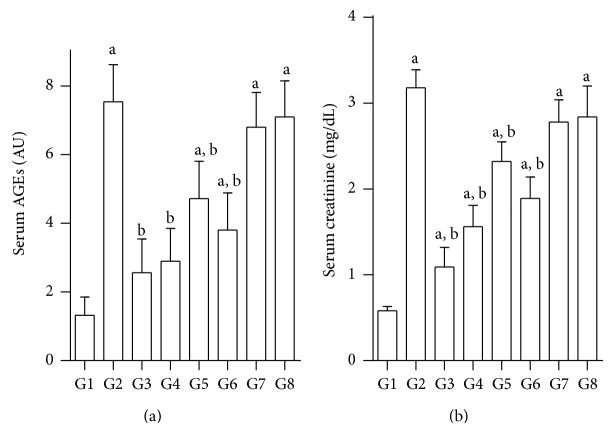
Effect of test compounds on serum AGEs and creatinine in nicotinamide, STZ diabetic rats; (a) serum advanced glycosylation end products (AGEs) measured as arbitrary units (AU); (b) serum creatinine (mg/dL); G1: normal control; G2: diabetic control (nicotinamide + STZ); G3: diabetic + glibenclamide (2.5 mg/kg); G4: diabetic + quercetin (10 mg/kg); G5: diabetic + JY-1; G6: diabetic + JY-2; G7: diabetic + JY-3; G8: diabetic + JY-4; (test compounds at optimised dose of 80 mg/kg); ^a^ *P* < 0.05 compared to normal rats (G1), ^b^ *P* < 0.05 compared to diabetic rats (G2) (one-way ANOVA followed by Tukey's post-hoc test).

There was a significant increase in AGEs and creatinine in diabetic rats (G1). Treatment with test compounds significantly normalized these values in all groups except for JY-3 and JY-4 groups. On the other hand, JY-2 treated animals showed significant lowering of AGEs and creatinine, which was comparable to both quercetin and glibenclamide (Figure 6).

### 3.7. Effect of Test Compounds on Oxidative Stress Markers and Liver Glycogen

#### 3.7.1. GSH

**Figure 7:**
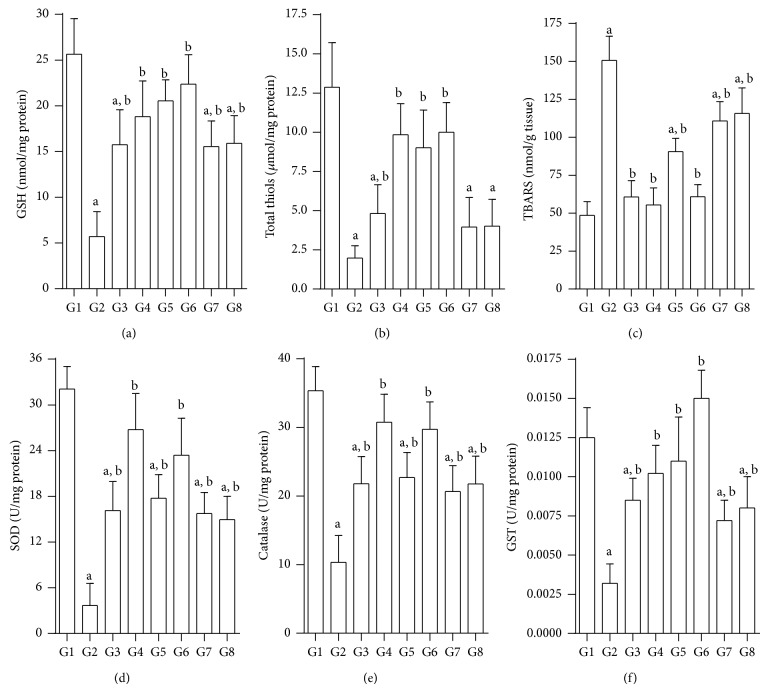
Effect of test compounds on enzymatic and nonenzymatic antioxidants and TBARS in nicotinamide, STZ diabetic rat liver; (a) glutathione (nmol/mg proteins); (b) total thiols (*μ*mol/mg protein); (c) TBARS (nmol/g of tissue); (d) SOD (U/mg protein); and (e) catalase (U/mg proteins); (f) GST (U/mg proteins); G1: normal control; G2: diabetic control (nicotinamide + STZ); G3: diabetic + glibenclamide (2.5 mg/kg); G4: diabetic + quercetin (10 mg/kg); G5: diabetic + JY-1; G6: diabetic + JY-2; G7: diabetic + JY-3; and G8: diabetic + JY-4; (test compounds at optimised dose of 80 mg/kg); ^a^ *P* < 0.05 compared to normal control (G1), ^b^ *P* < 0.05 compared to diabetic control (G2) (one-way ANOVA followed by Tukey's post-hoc test).

Basal GSH level of 25.7 ± 4.1 nmol/mg of protein (control group: G1) decreased significantly to 5.7 ± 2.7 nmol/mg of protein (G2) upon induction of diabetes (Figure 7(a)). Treatment with test compounds significantly increased GSH levels when compared to both diabetic controls (G2) and quercetin treated group. Interestingly, JY-2 was superior to glibenclamide in restoring GSH in diabetic rats.

#### 3.7.2. Total Thiols

Total thiol levels were significantly reduced upon induction of diabetes. However, test compounds produced a significant elevation in total thiols. JY-1 and JY-2 were found to be most effective in restoring total thiols in diabetic rats (Figure 7(b)). Glibenclamide and quercetin treatment could also restore total thiols significantly in a comparable manner.

#### 3.7.3. Lipid Peroxidation (TBARS)

TBARS increased by approximately threefold (48.62 ± 9.1 nmol/g in normal versus 150.7 ± 15.9 nmol in diabetic) in diabetic rats. On the other hand, test compounds lowered the TBARS significantly with JY-2 showing comparable efficacy to quercetin and glibenclamide (Figure 7(c)).

#### 3.7.4. SOD

Experimental diabetes produced SOD depletion by about 8-fold (3.7 ± 2.9 versus 32.1 ± 2.8 U/mg). Test compounds significantly elevated SOD levels with JY-1 showing an efficacy comparable to quercetin. JY-2, glibenclamide, and quercetin were found to have similar efficacy in restoring SOD in diabetic rats (Figure 7(d)).

#### 3.7.5. Catalase

Diabetic rats exhibited a threefold decrease in liver catalase levels (10.5 ± 3.9 U/mg versus 32.4 ± 3.6 U/mg of protein). On the other hand, test compounds significantly prevented the diabetes-induced decrease in catalase activity. Interestingly, quercetin was more effective than glibenclamide in restoring catalase activity in diabetic group (Figure 7(e)). JY-1 was comparable to glibenclamide in efficacy. On the other hand, JY-2 was comparable to quercetin and was more effective than glibenclamide.

#### 3.7.6. GST

The normal basal level of GST was found to be 0.0125 ± 0.002 U/mg of protein. In diabetic rats, GST was significantly decreased by about 4-fold (0.0033 ± 0.0014 U/mg). However, treatment with test compounds significantly elevated GST levels compared to untreated control diabetic rats. JY-1 and JY-2 restored GST levels significantly and was found to be superior to glibenclamide in efficacy. JY-2 was found to be even more effective than quercetin (Figure 7(f)).

#### 3.7.7. Liver Glycogen

**Figure 8:**
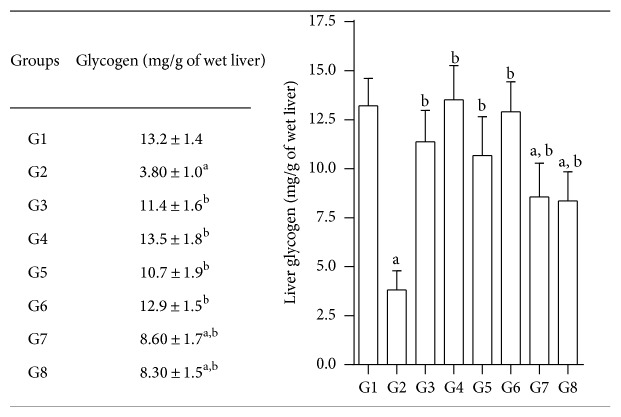
Effects of test compounds on liver glycogen levels in nicotinamide, STZ diabetic rats; values are in mg/g of wet liver (mean ± SEM); G1: normal control; G2: diabetic control (nicotinamide + STZ); G3: diabetic + glibenclamide (2.5 mg/kg); G4: diabetic + quercetin (10 mg/kg); G5: diabetic + JY-1; G6: diabetic + JY-2; G7: Diabetic + JY-3; and G8: diabetic + JY-4; (all test compounds at optimised dose of 80 mg/kg); ^a^ *P* < 0.05 compared to normal rats (G1); ^b^ *P* < 0.05 compared to diabetic rats (G2) (one-way ANOVA followed by Tukey's post-hoc test).

Diabetic rats showed significantly decreased levels of glycogen (3.80 ± 1.0 mg versus 13.2 ± 1.4 mg/g of wet liver) more than normal rats. However, treatment with test compounds significantly increased glycogen levels in diabetic rats. Quercetin also elevated the glycogen levels significantly and was more significant than glibenclamide in being able to raise liver glycogen (Figure 8 and Table inset).

### 3.8. Effect of Test Compounds on Pancreatic Tissue

**Figure 9:**
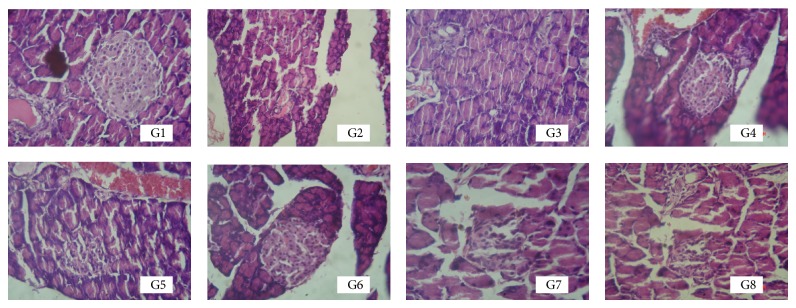
Histopathology of rat pancreas (40x); G1: normal control; G2: diabetic control (nicotinamide + STZ); G3: diabetic + glibenclamide (2.5 mg/kg); G4: diabetic + quercetin (10 mg/kg); G5: diabetic + JY-1; G6: diabetic + JY-2; G7: diabetic + JY-3; and G8: diabetic + JY-4; (all test compounds at optimised dose of 80 mg/kg).

The photographs of histology sections are depicted in Figure 9. Pancreas has very few number of ilet cells in nicotinamide-STZ rats (Figure 9, G2) compared to the normal animals (Figure 9, G1). Test compounds have protected the tissue damage and there was regeneration of pancreatic ilet cells. The protection was maximum in JY-2 followed by JY-1, JY-3, and JY-4 (Figure 9, G5–G8). Treatment with natural flavonoids, quercetin, showed maximum protection among all the treated rats and superior to glibenclamide (Figure 9, G4).

## 4. Discussion

Numerous animal models have been developed to understand the pathogenesis of diabetes. Many of them have been employed in screening of antidiabetic drugs. However, none of these models could reproduce complex pathology of human diabetes, especially T2D. Nicotinamide (120 mg/kg), when given prior to STZ (60 mg/kg) to rats, induces a diabetic with stable metabolic alterations and reduction in pancreatic insulin, effectively mimicing human T2D [12]. In the present study, we used STZ/nicotinamide diabetic rat model, with abnormal glucose tolerance and insulin activity to investigate the antidiabetic activity of test compounds.

STZ, when given alone, effectively induces type 1 diabetes in rats. The symptoms include severe glycaemia, glucosuria, polyphagia, polydipsia, and body weight loss, which occurs chiefly on account of loss of *β*-cells [20]. Quercetin stimulates the regeneration of *β*-cells in STZ rats by preventing *β*-cell apoptosis [20]. Many papers in published literature use this model for screening phytochemicals. However, if nicotinamide is injected prior to STZ, the severity of diabetes is attenuated to a certain extent, leading to a T2D like condition with insulin resistance [7, 8, 12]. In our present study we observed that the test compounds (benzopyrone analogues) are able to moderate FBG, AUC, and insulin levels in nicotinamide-STZ-diabetic rat model.

T2D is often linked with abnormal lipid metabolism, mainly because of insulin resistance. Impairment of insulin secretion will result in enhanced release of lipids from the adipose tissue into the plasma [7, 8]. There will be a variety of disorders in metabolic and regulatory pathways, which in turn lead to accumulation of lipids due to insulin deficiency [21]. Our studies revealed that supplementation with benzopyrone analogues showed significant attenuation in TG, TC, VLDL, and LDL. These effects could be because of reduced cholesterol biosynthesis or reduced rate of lipolysis. A high TC/HDL ratio is well known marker of dyslipidemia in cardiomyopathy and metabolic syndromes [7, 8]. Among the compounds synthesised, JY-2 was most effective in lowering lipids as well as dyslipidemic marker TC/HDL. Therefore, normalization of lipids in diabetic rats treated with the JY-2 may be partly due to increased insulin sensitivity at peripheral organs like skeletal muscles and fat cells and partly on account of increased insulin secretion by pancreatic *β*-cell regeneration. Compound JY-2 has restored the pancreatic *β*-cell integrity which is evident from the histopathological studies.

In T2D, a steady rise in insulin resistance leads to a corresponding increase in fasting insulin levels as the disease progresses. Increase in fasting insulin is one of the most important indications of insulin resistance in the present study. HOMA-IR is a parameter that can be calculated from serum insulin levels and is regarded widely as a reliable biomarker of insulin resistance. Test compounds such as JY-2 and JY-1 restored both insulin levels and HOMA-IR values to near normal.

STZ-induced diabetes also impairs cardiac function and kidney functions [22]. Increase in serum AGEs is one of the important indicators of these toxicities. Increased serum AGEs leads to a series of renal, neuronal, and blood vessel related toxicities that eventually lead to the microvascular complications in diabetes. Also, AGEs increases creatinine levels via renal toxicity [18]. In this study we have been able to successfully demonstrate the increase in AGEs in nicotinamide-STZ-induced diabetes. We were also able to establish that test compounds JY-1 and JY-2 effectively lowered AGEs and improved creatinine levels.

Hyperglycemia is a well-known cause for free radical generation, which in turn can stimulate lipid peroxidation (TBARS). Oxidation of excessive free fatty acids causes production of ROS, including H_2_O_2_, which inhibits glucose utilization by the tissue. The generation of ROS by hyperglycemia causes tissue GSH depletion. Decrease in GSH is a major reason for the harmful effects of lipid peroxidation. Diminished levels of enzymatic antioxidants such as SOD, catalase, and GST have been well documented in the diabetic condition [7, 8]. Treatment with compounds such as JY-2 combats the elevated levels of these enzymes, thereby indicating a protective effect.

There are reports on quercetin depleting GSH in animal studies and it is proposed because of the quercetin-quinone (QQ), a highly reactive radicle formation. The QQ-radicle formation is explained for quercetin and other compounds with the structures having hydroxyl groups at *β*-position and phenyl-3′, 4′-hydroxyl groups at *α*-position in *γ*-benzopyrones. None of the test compounds have hydroxyl group on phenyl ring at *α*-position and hence will not form QQ-radicle. The formation of QQ-radicle* in vivo* was debated in later reports. It is true in our studies also, we found quercetin and the test compounds restored the depleted GSH is to near normal in T2D-rats and test compounds are comparable to quercetin though the potency of test compounds is less than quercetin when doses are considered.

Glibenclamide monotherapy in humans at the initial months of treatment, increases fasting plasma insulin levels, and insulin responses to oral glucose challenges are increased. With chronic administration, circulating insulin levels decline to those that existed before treatment, but despite this reduction in insulin levels, reduced plasma glucose levels are maintained. The explanation relates to the fact that chronic hyperglycemia* per se* impairs insulin secretion (glucose toxicity in T2D). However many reported that animal models implicate the decline in fasting insulin level after glibenclamide treatment. Unlike glibenclamide, the test compounds did not show any hypoglycemia in normal rats. Therefore, we conclude that test compounds are hypoglycemic only in diabetic conditions. Further, antidiabetic activity of test compounds could have been mediated either by increasing the peripheral insulin sensitivity or by extrapancreatic mechanisms. It is also possible that the test compounds increase the survival of residual *β*-cell population, provoking an increase in insulin release. On the whole, we may conclude that treatment with these synthetic flavonoid analogues is corrective and not disruptive.

## 5. Conclusions

Test compounds JY-1, JY-2, JY-3, and JY4 showed significant antidiabetic activity in nicotinamide-STZ-induced diabetes in rats. Test compounds showed hypolipidemic activity along with the antidiabetic activity in diabetic rats. JY-2 exhibited maximum efficacy and was often equal or superior to standard drug glibenclamide. All test compounds significantly lowered oxidative stress in diabetic rats. The decrease in AGEs and restoration of creatinine levels have clinical relevance in preventing the cardiovascular and renal complications of diabetes.

## Figures and Tables

**Table 1:** Percentage reduction of glycemia by treatment with test compounds in nicotinamide-STZ diabetic rats.

Groups	Fasting blood glucose (FBG)		% Reduction of glycemia (taken from the mean values)
	Day 7	Day 22	
G1	90.2 ± 5.30	93.20 ± 9.40	−3.33
G2	188.3 ± 4.4	198.3 ± 12.3^a,b^	−5.31
G3	195.3 ± 6.6	108.3 ± 9.80^a,b,c^	44.55
G4	196.4 ± 3.5	114.2 ± 11.7^a,b,c^	41.86
G5	200.1 ± 8.7	145.2 ± 13.2^a,b,c^	27.44
G6	198.4 ± 3.7	132.6 ± 14.8^a,b,c^	31.15
G7	198.6 ± 3.8	168.8 ± 10.8^a,b,c^	15.01
G8	200.5 ± 2.9	173.2 ± 11.8^a,b,c^	13.62

All values are mean ± SEM; *n* = 6 (min); G1: normal control; G2: diabetic control (nicotinamide + STZ); G3: diabetic + glibenclamide (2.5 mg/kg); G4: diabetic + quercetin (10 mg/kg); G5: diabetic + JY-1; G6: diabetic + JY-2; G7: diabetic + JY-3; and G8: diabetic + JY-4; (test compounds at optimised dose of 80 mg/kg); ^a^ *P* < 0.05 FBG on day 22 is compared to FBG on day 7; ^b^ *P* < 0.05 compared to normal rats (G1); and ^c^ *P* < 0.05 is compared to diabetic rats (G2) (one-way ANOVA followed by Tukey's post-hoc test).

## Abbreviations

AGEs: Advanced glycation end products
AUC: Area under the curve
BSA: Bovine serum albumin
CDNB: 1-Chloro-2,4-dinitrobenzene
CMC: Carboxymethyl cellulose
DAG: Diacylglycerol
DM: Diabetes mellitus
DTNB: 5,5′-dithio-bis-2-nitrobenzoic acid
FBG: Fasting blood glucose
GSH: Reduced glutathione
GST: Glutathione-s-transferase
HDL: High density lipoproteins
HOMA-IR: Homeostatic model assessment
ITT: Insulin tolerance test
LDL: Low density lipoproteins
OGTT: Oral glucose tolerance test
PKC: Protein kinase C
ROS: Reactive oxygen species
SOD: Superoxide dismutase
STZ: Streptozotocin
T2D: Type 2 diabetes
TBA: Thiobarbituric acid
TBARS: Thiobarbituric acid reactive substances
TC: Total cholesterol
TG: Triglycerides
VLDL: Very low density lipoproteins.

## References

[1] Poitout V., Robertson R. P. (2008). Glucolipotoxicity: fuel excess and *β*-cell dysfunction. *Endocrine Reviews*.

[2] Brownlee M. (2005). The pathobiology of diabetic complications: a unifying mechanism. *Diabetes*.

[3] Brownlee M. (2001). Biochemistry and molecular cell biology of diabetic complications. *Nature*.

[4] Calcutt N. A., Cooper M. E., Kern T. S., Schmidt A. M. (2009). Therapies for hyperglycaemia-induced diabetic complications: from animal models to clinical trials. *Nature Reviews Drug Discovery*.

[5] Nayak Y., Jayashree B. S., Unnikrishnan M. K. (2012). Benzopyran-4-one is an ideal pharmacophore for lead optimization in antidiabetic drug discovery. *Research Journal of Pharmacy and Technology*.

[6] Cazarolli L. H., Zanatta L., Alberton E. H., Figueiredo M. S. R. B., Folador P., Damazio R. G., Pizzolatti M. G., Silva F. R. M. B. (2008). Flavonoids: cellular and molecular mechanism of action in glucose homeostasis. *Mini-Reviews in Medicinal Chemistry*.

[7] Veerapur V. P., Prabhakar K. R., Kandadi M. R., Srinivasan K. K., Unnikrishnan M. K. (2010). Antidiabetic effect of Dodonaea viscosa aerial parts in high fat diet and low dose streptozotocin-induced type 2 diabetic rats: a mechanistic approach. *Pharmaceutical Biology*.

[8] Veerapur V. P., Prabhakar K. R., Thippeswamy B. S., Bansal P., Srinivasan K. K., Unnikrishnan M. K. (2010). Antidiabetic effect of *Dodonaea viscosa* (L). Lacq. aerial parts in high fructose-fed insulin resistant rats: a mechanism based study. *Indian Journal of Experimental Biology*.

[9] Havsteen B. H. (2002). The biochemistry and medical significance of the flavonoids. *Pharmacology & Therapeutics*.

[10] Jayashree B. S., Noor F. A., Yogendra N., Vijay Kumar D. (2008). Synthesis of substituted 3-hydroxy flavones for antioxidant and antimicrobial activity. *Pharmacologyonline*.

[11] Nayak Y., Jayashree B., Unnikrishnan M. Antioxidant, DNA protection and antiglycation activity of benzopyrone-4-one analogues.

[12] Punitha I. S. R., Rajendran K., Shirwaikar A. (2005). Alcoholic stem extract of Coscinium fenestratum regulates carbohydrate metabolism and improves antioxidant status in streptozotocin-nicotinamide induced diabetic rats. *Evidence-Based Complementary and Alternative Medicine*.

[13] Nayak Y., Venkatachalam H., Vijay Kumar D. Synthesis of benzopyran-4-one analogues and their antidiabetic activity in insulin resistant rats.

[14] Rossini A. A., Williams R. M., Appel M. C., Like A. A. (1978). Sex differences in the multiple-dose streptozotocin model of diabetes. *Endocrinology*.

[15] Friedewald W. T., Levy R. I., Fredrickson D. S. (1972). Estimation of the concentration of low-density lipoprotein cholesterol in plasma, without use of the preparative ultracentrifuge.. *Clinical Chemistry*.

[16] Matthews D. R., Hosker J. P., Rudenski A. S., Naylor B. A., Treacher D. F., Turner R. C. (1985). Homeostasis model assessment: insulin resistance and *β*-cell function from fasting plasma glucose and insulin concentrations in man. *Diabetologia*.

[17] Akinmokun A., Selby P. L., Ramaiya K., Alberti K. G. M. M. (1992). The short insulin tolerance test for determination of insulin sensitivity: a comparison with the euglycaemic clamp. *Diabetic Medicine*.

[18] Yanagisawa K., Makita Z., Shiroshita K. (1998). Specific fluorescence assay for advanced glycation end products in blood and urine of diabetic patients. *Metabolism: Clinical and Experimental*.

[19] vn der Vies J. (1954). Two methods for the determination of glycogen in liver. *The Biochemical Journal*.

[20] Coskun O., Kanter M., Korkmaz A., Oter S. (2005). Quercetin, a flavonoid antioxidant, prevents and protects streptozotocin-induced oxidative stress and *β*-cell damage in rat pancreas. *Pharmacological Research*.

[21] Goldberg R. B. (1981). Lipid disorders in diabetes. *Diabetes Care*.

[22] Arozal W., Watanabe K., Veeraveedu P. T., Ma M., Thandavarayan R. A., Suzuki K., Tachikawa H., Kodama M., Aizawa Y. (2009). Effects of angiotensin receptor blocker on oxidative stress and cardio-renal function in streptozotocin-induced diabetic rats. *Biological and Pharmaceutical Bulletin*.

